# Effect of PAR-2 Deficiency in Mice on KC Expression after Intratracheal LPS Administration

**DOI:** 10.1155/2011/415195

**Published:** 2011-12-07

**Authors:** Julie C. Williams, Rebecca D. Lee, Claire M. Doerschuk, Nigel Mackman

**Affiliations:** ^1^Division of Hematology and Oncology, Department of Medicine, McAllister Heart Institute, The University of North Carolina at Chapel Hill, Chapel Hill, NC 27599-7035, USA; ^2^Department of Medicine, Center for Airways Disease, The University of North Carolina at Chapel Hill, Chapel Hill, NC 27599-7035, USA

## Abstract

Protease activated receptors (PAR) have been shown to play a role in inflammation. PAR-2 is expressed by numerous cells in the lung and has either proinflammatory, anti-inflammatory, or no effect depending on the model. Here, we examined the role of PAR-2 in a model of LPS-induced lung inflammation. We found that PAR-2-deficient mice had significantly less KC expression in bronchial lavage fluid compared with wild-type mice but there was no difference in MIP-2 or TNF-**α** expression. We also found that isolated alveolar and resident peritoneal macrophages lacking PAR-2 showed a similar deficit in KC after LPS stimulation without differences in MIP-2 or TNF-**α**. Infiltration of neutrophils and macrophages into the lung following LPS administration was not affected by an absence of PAR-2. Our results support the notion that PAR-2 plays a role in LPS activation of TLR4 signaling in macrophages.

## 1. Introduction

The protease activated receptors (PAR) are a four-member family of 7 transmembrane G-protein-coupled receptors that are activated by cleavage of an amino terminal sequence resulting in exposure of a tethered ligand. PARs act as sensors of both coagulation and noncoagulation proteases, including thrombin, trypsin, and mast cell tryptase. It is thought that PARs act as a major link between coagulation and inflammation. PARs are expressed in many different cell types. In the lung, PAR-2 is expressed by epithelial cells [1], smooth muscle cells [1], fibroblasts [2], and endothelial cells [3]. In addition, PAR-2 is expressed on a variety of immune cells including mast cells [4], macrophages [5], and neutrophils [6]. 

PAR-2 is activated by trypsin, mast cell tryptase, matriptase, and the coagulation proteases Factor VIIa and Xa *in vitro *[7]. However, it is unclear which proteases activate PAR-2 *in vivo *[7]. Mimics of the tethered ligand, termed PAR-2 agonist peptides (PAR-2 AP), can also induce PAR-2 signaling. Scrambled analogs to these peptides are often used as negative controls. However, signaling initiated by proteases and agonist peptides may not have the same results [8]. 

All 4 PARs have been shown to be present in rat lungs, and their expression is modulated after endotoxin administration [9]. The role of PAR-2 in lung inflammation is controversial. PAR-2 has been found to be upregulated in the lung following exposure to viruses, lipopolysaccharide (LPS), tobacco smoke, and allergens [10, 11]. Two groups found that administration of large amounts of PAR-2 AP in mouse airways reduced cellular infiltration induced by LPS [12, 13]. This reduction may be related to PAR2-dependent production of prostaglandin E2 [13]. However, another group found no differences in three different models of acute lung injury between PAR-2-deficient and wild-type mice [14]. A proinflammatory role for PAR-2 in the lung has also been observed in PAR-2-deficient mice by two different groups [15, 16]. These contradictory observations may be due to differing administration routes of the agonist peptide, the amounts used, and the type of insult. Clearly, further investigation into the role of PAR-2 in lung inflammation is warranted. 

It has been proposed that PAR-2 and the LPS receptor, toll-like receptor 4 (TLR4) cooperate to enhance proinflammatory signaling [17]. This group found that cotransfection of HEK293T cells with PAR-2 and TLR4 enhanced NF-*κ*B activation by PAR-2 AP. This effect was specific to TLR4, as TLR3 and TLR2 did not show similar enhancement [17]. It was also noted that the TLR4-mediated enhancement was working through the intracellular adaptor molecules TRIF and TRAM [17]. However, more recent work by the same group shows that stimulation of thioglycollate elicited peritoneal macrophages with PAR-2 AP suppressed proinflammatory cytokines and enhanced anti-inflammatory cytokine production in response to LPS [18]. In addition, thioglycollate-elicited peritoneal macrophages from PAR-2-deficient mice were found to have a hyperinflammatory response to LPS [18]. Therefore, the PAR-2 and TLR4 signaling responses are likely intertwined though the exact mechanism and *in vivo* relationship have yet to be elucidated. 

In order to further elucidate the role of PAR-2 in LPS induced lung inflammation, we subjected PAR-2-deficient and wild-type mice to intratracheal LPS administration. We found no difference in cellular infiltration into the lungs. We observed a deficit in the chemokine, keratinocyte chemoattractant (KC; CXCL1), in the bronchial alveolar lavage fluid (BALF) from PAR-2-deficient mice. In addition, PAR-2 deficiency had no effect on the proinflammatory cytokine tumor necrosis factor-*α* (TNF-*α*), or the chemokine macrophage inflammatory protein-2 (MIP-2; CXCL2) in the BALF. However, compared to wild-type mice, MIP-2 levels were found to be lower in lung homogenates of PAR-2-deficient mice treated with LPS. Furthermore, we found that PAR-2-deficient alveolar and resident peritoneal macrophages produced less KC after *ex vivo* LPS stimulation. 

## 2. Materials and Methods

### 2.1. Mice

The generation of PAR-2^+/+^ (wild-type) and PAR-2^−/−^ mice has been previously described [19]. Mice were 8 to 10 weeks of age at the time of experiments. All experimental protocols were approved by the University of North Carolina-Chapel Hill's Institutional Animal Care and Use Committee. 

### 2.2. Intratracheal LPS Instillation and BALF Collection

The method of intratracheal LPS instillation has been described [20]. Mice were anesthetized by intraperitoneal injection of 12.5 mg/mL tribromoethanol (TBE) (Acros Organics), at a dose of 0.02 mL TBE per gram of mouse body weight. LPS from *E. coli *serotype O111:B4 was purchased from Sigma-Aldrich. 10 *μ*g of LPS was instilled into the left lung of wild-type or PAR-2 knockout mice, and animals were sacrificed at indicated timepoints by diaphragmatic incision. Control mice did not receive LPS. Lungs from treated and untreated mice were lavaged postmortem by insertion of a 27-gauge catheter into the exposed trachea (BD Biosciences). The lungs were instilled three times with 900 *μ*L of phosphate buffered saline  (PBS), and the BALF was suctioned out of the lungs after each instillation using a 1 mL syringe. 

### 2.3. Sample Preparation

BALF was prepared for ELISA by immediate centrifugation of 1 mL samples at 500 ×g for 20 minutes at 4°C, and the supernatant was frozen and stored at −80°C. Pelleted cells were resuspended in 200 *μ*L PBS and retained for evaluation by flow cytometry. After BALF collection, the left lung lobe was excised, snap frozen in liquid nitrogen, and stored at −80°C prior to homogenization. For protein extraction, the lungs were thawed, weighed, and suspended in lysis buffer (1% SDS, 10% Glycerol, and 100 mM Tris) with protease (Sigma-Aldrich) and phosphatase inhibitors (Thermo Scientific). One hundred *μ*L of lysis buffer per 10 mg of tissue was used. Lung tissue was ground with a homogenizer for ~1 minute, and the samples were rested on ice for 30 minutes before centrifugation at 4°C, 16,000 ×g for 10 minutes. The protein concentration in the samples was measured using the DC Protein Assay from Bio-Rad Laboratories, and the homogenate was aliquoted and frozen at −80°C until use. 

### 2.4. ELISA

Mouse KC, MIP-2, Lix, and TNF-*α* DuoSet ELISA kits were purchased from R&D Systems. 

### 2.5. Flow Cytometry

Cells were collected from BALF as described in sample preparation. Total non-red blood cells were then enumerated using a Coulter counter (Beckman Coulter). Cells were stained as previously described [21] with anti-mouse F4/80 Pacific Blue and anti-mouse 7/4-FITC, both purchased from AbD Serotec (Oxford, UK). 

### 2.6. LPS Stimulation of Macrophages

For alveolar macrophages, cells were isolated from individual mice as described in sample preparation. Resident peritoneal macrophages were harvested as previously described [21]. Cells were then counted and plated in 150 *μ*L of media (DMEM-H containing 10% FBS and Penicillin/Streptomycin) in 96 well plates. After 3 hours, nonadherent cells were removed. The next day cells were stimulated for indicated times with 100 ng/mL of LPS. 

## 3. Results

### 3.1. Mice Lacking PAR-2 Have Reduced KC Expression Following Intratracheal LPS Instillation

**Figure 1:**
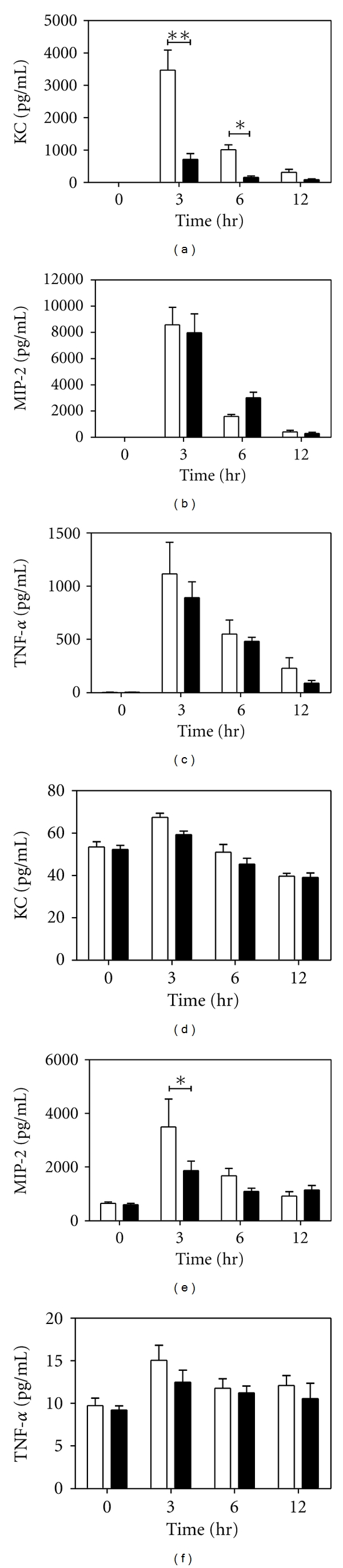
Chemokine and cytokine expression in BALF and lung homogenates after intratracheal LPS instillation. 10 *μ*g of LPS (*E. coli *O111:B4) was instilled into the left lungs of wild-type (PAR2^+/+^, white bars) and PAR-2^−/−^ (black bars) mice for indicated time periods. BALF and lungs were collected, and lungs were homogenized. ELISAs on BALF were performed for KC (a), MIP-2 (b), and TNF-*α* (c). ELISAs on lung homogenates were performed for KC (d), MIP-2 (e), and TNF-*α* (f). *n* ≥ 4. *P < 0.05; **P < 0.001 by two way ANOVA posttest.

In order to determine the effects of PAR-2 during acute lung inflammation, we instilled LPS into the left lung of wild-type and PAR-2^−/−^ mice. We then harvested BALF and lung tissue and performed ELISAs for the chemokines KC, MIP-2, and Lix, and the proinflammatory cytokine TNF-*α*. As shown in Figure 1(a), BALF collected from mice lacking PAR-2 had significantly reduced KC at 3 and 6 hours after LPS instillation. However, MIP-2, TNF-*α*, and Lix levels in the BALF were unaffected by PAR-2 deficiency (Figures 1(b) and 1(c), data not shown). Surprisingly, KC levels in lung homogenates from mice in Figure 1(a) showed little KC induction and no difference between genotypes (Figure 1(d)). A transient deficit in MIP-2 was found at 3 hours after LPS instillation in mice lacking PAR-2, although this deficit was not apparent at the 6 and 12 hour time points (Figure 1(e)). Finally, TNF-*α* showed a small increase in lung homogenates 3 hours after LPS instillation; however, no differences were observed between genotypes (Figure 1(f)).

### 3.2. Alveolar and Resident Peritoneal Macrophages Lacking PAR-2 Have Reduced KC Expression Following LPS Stimulation

**Figure 2:**
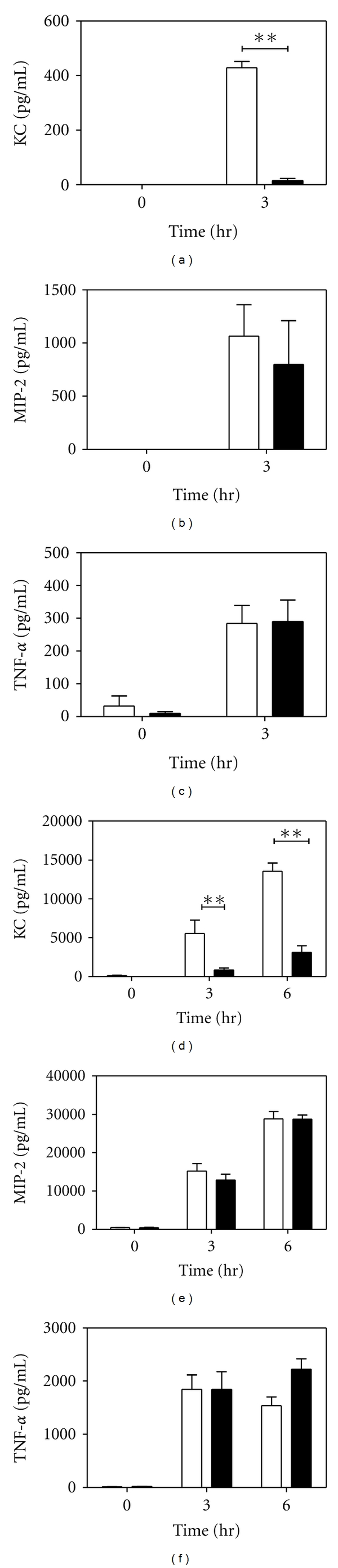
LPS stimulation of chemokines and TNF-*α* in alveolar and resident peritoneal macrophages. Macrophages from wild-type (PAR2^+/+^, white bars) and PAR-2^−/−^ (black bars) mice were left untreated or stimulated with 100 ng/mL of LPS for indicated time periods. ELISAs on cell supernatants from alveolar macrophages were performed for KC (a), MIP-2 (b), and TNF-*α* (c). ELISAs on cell supernatants from resident peritoneal macrophages were performed for KC (d), MIP-2 (e), and TNF-*α* (f). *n* = 3. *P < 0.05; **P < 0.001 by two way ANOVA posttest.

Since KC production was found to be dramatically elevated in BALF compared to lung homogenates and the PAR-2-dependent KC deficit was only observed in BALF, we hypothesized that alveolar macrophages may be the source of KC in the BALF. Therefore, we isolated alveolar macrophages from naïve wild-type and PAR-2-deficient mice and stimulated them with LPS for 3 hours because the largest amount of KC in the BALF was observed 3 hours after LPS instillation. We observed a significant deficit in KC production by alveolar macrophages isolated from PAR-2^−/−^ animals compared to their wild-type counterparts (Figure 2(a)). Similar to Figure 1, we found no significant differences between genotypes in MIP-2 or TNF-*α* levels in alveolar macrophage cell supernatants (Figures 2(b) and 2(c)). Since only a small number of alveolar macrophages can be isolated, we repeated a similar experiment using resident peritoneal macrophages stimulated with LPS for 3 and 6 hours. We observed a significant deficit in KC expression at 3 and 6 hours in cells from mice lacking PAR-2 (Figure 2(d)). Although MIP-2 and TNF-*α* were dramatically increased following LPS stimulation, we found no differences between genotypes in MIP-2 or TNF-*α* expression by resident peritoneal macrophages (Figures 2(e) and 2(f)). 

### 3.3. No Effect on Cellular Infiltration to LPS Instilled Lungs in PAR-2-Deficient Mice

**Figure 3:**
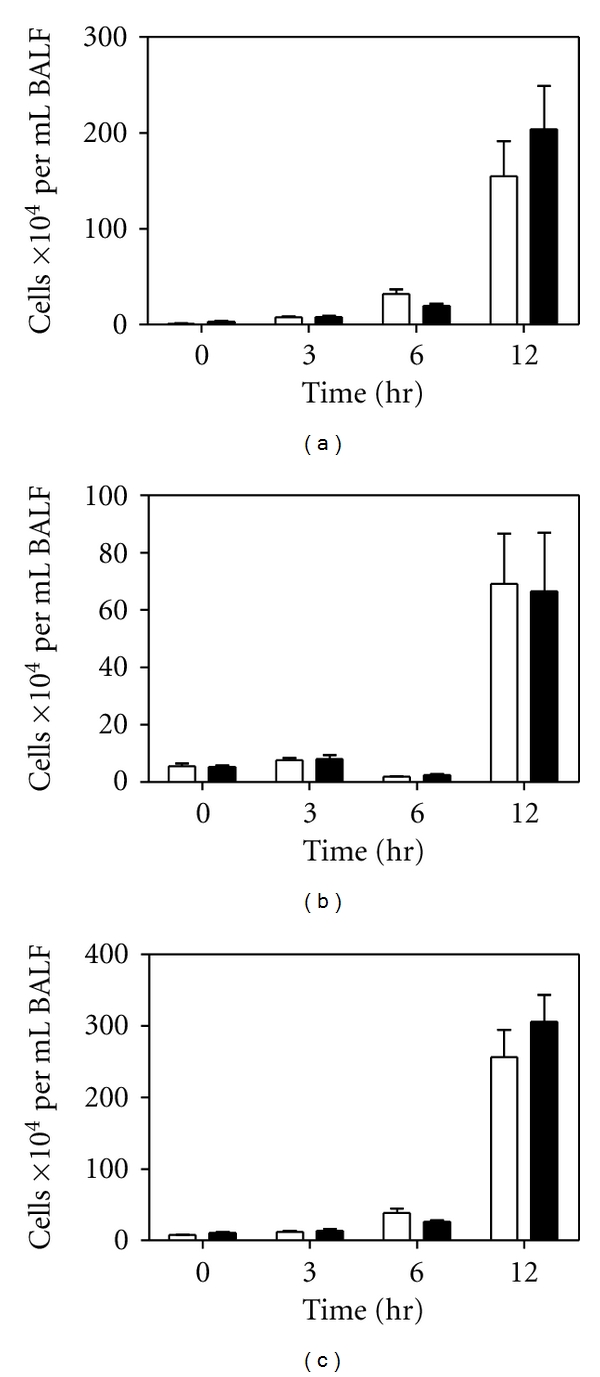
Cellular infiltration into the lung following LPS instillation. BALF was collected from wild-type (PAR2^+/+^, white bars) and PAR-2^−/−^ (black bars) mice at indicated time periods after intratracheal LPS instillation. Neutrophils (a), macrophages (b), and total cells (c) were enumerated by flow cytometry. *n* ≥ 4.

In order to determine if the observed deficit in KC expression in BALF and alveolar macrophages resulted in a deficit in cellular infiltration, we isolated cells from the BALF following LPS instillation. We observed neutrophil and macrophage infiltration by flow cytometry. We found no significant differences in neutrophil (Figure 3(a)), macrophage (Figure 3(b)) or total cellular (Figure 3(c)) infiltration in the BALF of PAR-2^−/−^ mice compared to their wild-type counterparts. 

## 4. Discussion

Here, we have presented data showing that a lack of PAR-2 leads to a deficit in KC expression both *in vivo* and *in vitro*. This deficit is specific to KC as other chemokines including MIP-2 and Lix (data not shown) were similar between genotypes. In addition, the proinflammatory cytokine TNF-*α* is not affected by the absence of PAR-2 *in vivo* or *in vitro*. Interestingly, we found similar levels of KC in the lung tissue in both genotypes; in fact, little induction of KC was observed in the lung homogenates after LPS administration. This suggests that the cells found within the BALF, such as alveolar macrophages, are the major source of KC in the lung in response to intratracheal LPS stimulation. In contrast, MIP-2 was found to be induced in both the BALF and lung tissues. While MIP-2 in the BALF was induced by LPS, no differences were found between genotypes. However, a transient deficit in MIP-2 induction was observed in the lung homogenate of animals lacking PAR-2 3 hours after intratracheal LPS instillation. This data suggests that cells other than the alveolar macrophages use PAR-2 for MIP-2 expression, though the relative contribution of alveolar macrophages and other cells in the lung to MIP-2 production is still unclear.

Although deficits in KC induction in both mice and cells lacking PAR-2 were dramatic, the biological relevance of this pathway remains to be elucidated. It is well known that KC is a potent chemokine that recruits neutrophils and macrophages to sites of infection. However, we did not observe a difference in cellular infiltration into the lungs of animals lacking PAR-2, though cellular infiltrates significantly increased in both genotypes. It is possible that the production of other chemokines which recruit neutrophils and macrophages, such as MIP-2 and Lix, is sufficient. 

We also observed a deficit in KC expression in LPS-treated resident peritoneal macrophages lacking PAR-2, suggesting that PAR-2 signaling is required for KC expression by macrophages in locations other than the lung. Similar to alveolar macrophages, MIP-2 and TNF-*α* production was unaffected by the lack of PAR-2 in resident peritoneal macrophages. Interestingly, Peters and colleagues found that costimulation of alveolar macrophages with LPS and PAR-2 AP showed similar induction of MIP-2 *in vitro* compared to LPS alone [13]. Similarly, we found that PAR-2 AP was unable to stimulate KC or MIP-2 production by alveolar macrophages (data not shown). In addition, KC and MIP-2 production by alveolar macrophages costimulated with LPS and PAR-2 AP was similar to stimulation with LPS alone (data not shown). Taken together, these data suggest that signaling via PAR-2 resulting in chemokine production may not require PAR-2 activation.

Other groups have investigated a role for PAR-2 in lung inflammation. However, the use of knockout mice has been limited and, surprisingly, few have examined chemokine and proinflammatory cytokine production. Large amounts of PAR-2 AP have also been used to reduce cellular infiltration induced by LPS administration in the lungs [12]. However, the mechanism of this suppression was not determined and importantly was not shown to be absent in PAR-2^−/−^mice. Another group found similar reductions in cellular infiltration into the lung using large amounts of PAR-2 AP and LPS coadministration compared to LPS alone. However, this group found a deficit in KC and MIP-2 in the BALF 1 hour after stimulation, although this deficit was no longer apparent after 3 hours [13]. These results are in contrast to the findings presented in this paper; however, both of these groups used Balb/C mice and LPS from *E. coli* strain 0127:B8. It is possible that mouse and LPS strain differences contribute to this discrepancy. 

It is also important to note that the proteases that activate PAR-2 in the lung have not been well defined. It is speculated that mast cell tryptase is the endogenous activator of PAR-2 in the lung. However, it is possible that other, as yet unidentified, enzymes contribute to PAR-2 cleavage. In addition, the activation of PAR-2 by the PAR-2 AP and the endogenous tethered ligand may result in different downstream signaling events [8, 22]. Further investigation of the role of PAR-2 in lung inflammation is critical, as blockade of PAR-2 has been suggested as a possible therapeutic approach to reduce lung inflammation. Importantly, the inhibition of PAR-2 during chronic or allergic lung inflammation may have entirely different results from the acute lung inflammatory states presented in this study.
